# Both evenness and dominant species identity have effects on litter decomposition

**DOI:** 10.1002/ece3.11052

**Published:** 2024-02-26

**Authors:** Baijie Fan, Ziqing Gong, Xiaojing Xin, Yulin Liu, Luoyang He, Yubao Gao, Anzhi Ren, Nianxi Zhao

**Affiliations:** ^1^ Department of Plant Biology and Ecology, College of Life Science Nankai University Tianjin China

**Keywords:** decomposition process, initial litter functional structure, litter quality, relative abundance, relative mixture effect

## Abstract

Exploring how interactions between species evenness and dominant species identity affect litter decomposition processes is vital to understanding the relationship between biodiversity and ecosystem functioning in the context of global changes. We carried out a 127‐day litter decomposition experiment under controlled conditions, with interactions of four species evenness types (high, medium, low and single species) and three dominant species identity (*Leymus chinensis*, *Serratula centauroides*, *Artemisia capillaris*). After collecting the remaining litter, we estimated how evenness and dominant species identity affected litter mass loss rate, carbon (C) loss rate, nitrogen (N) loss rate and remaining litter C/N directly or indirectly, and assessed relative mixture effects (RMEs) on litter mass loss. The main results are shown as follows. (1) By generalized linear models, litter mass loss rate was significantly affected by evenness after 69‐day decomposition; N loss rate was affected by dominant species identity after 69‐day decomposition, with treatment dominated by *Serratula centauroides* being at least 9.26% higher than that dominated by any of other species; and remaining litter C/N was affected by the interactions between evenness and dominant species identity after 30‐, 69‐ and 127‐day decomposition. (2) Twenty‐three out of 27 RMEs were additive, and dominant species identity showed a significant effect on RMEs after 127‐day decomposition. (3) By confirmatory path analyses, litter mass loss rate was affected by dominant species identity directly after 127‐day decomposition, and by both species evenness and dominant species identity indirectly which was mediated by initial litter functional dispersion (FDis) after 30‐ and 69‐day decomposition; remaining litter C/N was affected by evenness indirectly which was mediated by initial litter FDis after 127‐day decomposition. These findings highlight the importance of evenness and dominant species identity on litter decomposition. The study provides insights into communities during retrogressive successions in semi‐arid grasslands in the context of global changes.

## Graphical abstract

This study emphasized the importance of evenness and dominant species identity in affecting litter decomposition rate and provide theoretical significance for predicting community nutrient cycles under the condition of changes in community structure in semi‐arid grasslands.

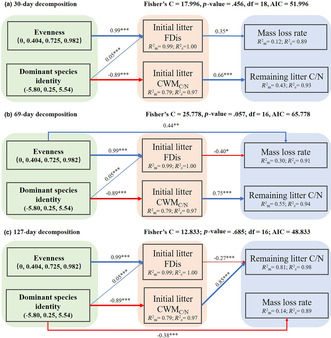

## 1 INTRODUCTION

In the context of global climatic change and human disturbance, species abundance patterns would shift towards higher dominance (Dangles & Malmqvist, 2004; Tilman et al., 2001), which may affect litter species composition and relative abundance and consequently ecosystem functioning. In early studies, most biodiversity‐ecosystem functioning (BEF) experiments have focused on the effects of species richness, that is, biodiversity has been reduced to a single number with equal species proportion (Hubbell, 2001), which involves higher species evenness than is encountered in nature (Wardle, 2013). With the development of BEF, ecologists demonstrate that the shape of richness‐ecosystem functioning relationship can be predicted by the dominance/evenness status of communities, and stress the importance of dominant species identity as a fundamental component of diversity (Dangles & Malmqvist, 2004).

Litter decomposition processes, such as litter decomposition rates and remaining litter quality, are very important for ecosystem functioning, including plant productivity and ecosystem nutrient cycles (Bradford et al., 2016; Liski et al., 2003; Taylor et al., 2007). Different litters usually show different qualities which are related to decomposition biotic communities such as soil microbial community, resulting in different decomposition rates (Setiawan et al., 2016). Thus, considering litter species‐specific interactions at intra‐ and interspecific levels, both biotic and abiotic decomposition environments under single‐species litter conditions would be different from those under multi‐species litter mixture conditions. As a result, litter decomposition processes would be associated with litter species composition and species relative abundance. Therefore, exploring how changes of litter species composition and species relative abundance affect decomposition processes and the underlying mechanisms is necessary to further understand the ecological consequences of community dynamics (Liu et al., 2016; Pires et al., 2018); moreover, such knowledge is very important for the development of BEF relationship.

A growing number of studies have found that litter species evenness is very important in regulating litter decomposition processes, including litter decomposition constant (Lin et al., 2013), litter mass loss (Swan et al., 2009) and litter nitrogen (N) loss (Zhang et al., 2022). Meanwhile, several studies have shown that litter species with the highest abundance (dominant species) within uneven mixed litter treatments can significantly affect litter mass loss rates when the qualities of used litter are significantly different (McLaren & Turkington, 2011; Migliorini et al., 2018). Therefore, researchers have recently focused on how dominant species identity interacts with the effect of evenness on litter decomposition characteristics. However, previous studies have demonstrated that the interactions between evenness and dominant species identity on litter mass loss are not significant (Dickson & Wilsey, 2009; Li et al., 2013), and the underlying mechanisms remain elusive. Considering that change in resource complementarity, the species‐specific reactions and interspecific interactions of litter mixtures often differ due to the litter characteristics of dominant species and litter species evenness (Bonanomi et al., 2010). Therefore, deepening understanding of their interactive effect on litter decomposition processes and exploring the mechanisms of how they regulate litter decomposition in mixtures are important for us to further understand the ecological consequences of changes of community species (especially for the dominant species) and their relative abundance during community succession processes facing global changes.

Relative mixture effects (RMEs) are usually used to estimate the mechanism of litter mixtures (multispecies litter) on decomposition processes. Considering the significantly different biotic and abiotic decomposition environments between single‐species litter and litter in mixtures (multi‐species litter), the observed decomposition rates in mixtures are probably different from the expected ones which are calculated based on single‐species litter decomposition by assuming additive mixture effect (Chen et al., 2021; Guo et al., 2020). In other words, RMEs might be shown as non‐additive effects which include synergistic effects (positive RMEs) and antagonistic effects (negative RMEs). Furthermore, with the development of trait‐based functional ecology, community functional structure, including community‐weighted mean trait values (CWM) and functional dispersion (FDis), can be calculated easily based on the species traits and species relative abundance (SRA) in a community (Diaz et al., 2007), which makes it easy to explore the mechanisms by which the litter species composition and relative abundance affect litter decomposition processes from the viewpoint of functional ecology. CWM is calculated as the community‐level trait value weighted by SRA; and a significant positive CWM‐ecosystem functioning relationship supports the mass ratio hypothesis (Grime, 1998), which emphasizes the importance of dominant species on ecosystem functioning (Zeng et al., 2020). FDis is the mean distance of individuals in single or multi‐dimensional trait space to the centroid of all species via the weighting distance of individual species by its SRA (Laliberte & Legendre, 2010). The significant positive FDis‐ecosystem functioning relationship supports the niche complementarity hypothesis (Tilman et al., 1997), which emphasizes the importance of trait differences among different species on ecosystem functioning (Loreau & Hector, 2001). Both CWM and FDis are sensitive to the changes in traits of dominant species and SRA, which can reflect community functional responses to community composition and structure changes during environmental variations (Tobner et al., 2016). Although many studies have explored the effects and relative significance of CWM and FDis on soil ecosystem functioning or productivity, only a few studies have focused on their effects or relative importance on litter decomposition processes (Li et al., 2022; Zhang et al., 2020a), and even fewer studies have focused on how CWM and FDis mediate the response of litter decomposition to changes in litter species evenness or dominant species identity (Zhang et al., 2020a). Therefore, the current understanding of the mechanism of how initial litter composition affects litter decomposition processes is incomplete, which weakens the ability to quantify and predict ecosystem functioning responses to community changes.

In recent years, ecosystem functioning in arid and semi‐arid grassland has significantly changed due to community changes caused by global climate change and human activities (Jiang et al., 2013). For example, the communities in Inner Mongolia grassland of China, which is an essential part of the European‐Asian grassland, have been experiencing community retrogressive succession (Cheng et al., 2013; Jiang et al., 2022). Especially, in Hulun Buir Steppe, *Leymus chinensis* is a zonally dominant species (relative mass is higher than 30%), with a forb *Serratula centauroides* and a semi‐shrub *Artemisia capillaris* being accompanied species (relative mass is lower than 5%). However, *S. centauroides* and *A. capillaris* have taken the place of *L. chinensis* and become dominant species in some areas because of the increasing climate aridity and human activities in recent decades, with the decreasing relative mass of *L. chinensis* (lower than 10% in some area) (Xu et al., 2014). Therefore, even in a narrow region in Hulun Buir Steppe, communities (or large‐area patches) dominated by *L. chinensis*, *S. centauroides* or *A. capillaris*, can be found from one place to another; consequently, litter mixtures, as well as community species composition and SRA, are significantly different within these three communities (or large‐area patches). How such changes in litter species evenness and dominant species identity affect decomposition processes and the underlying mechanisms remain unknown, and the gap of knowledge may limit our understanding of nutrient cycling in grassland ecosystems under the conditions of rapid community succession.

In the present study, the litter of three species in Hulun Buir Steppe mentioned above was collected in a natural community, that is, *L. chinensis*, *S. centauroides* and *A. capillaris*, and they often co‐exist within communities. We carried out a two‐factor experiment with species evenness (high, medium, low and single species) and dominant species identity (*L. chinensis*, *S. centauroides* or *A. capillaris*) using the litter of these three species to explore how evenness and dominant species identity and their interactions affected litter decomposition processes and the underlying mechanisms. Previous studies have shown that litter C loss rate and N loss rate as well as litter mass loss rate are important indicators reflecting litter decomposition rate and that remaining litter C/N has an instructive effect on remaining litter quality (Wardle, 2013; Yang et al., 2012); thus, litter mass loss rate, C loss rate, N loss rate and the remaining litter C/N were calculated in this study. RMEs on mass loss were estimated, and initial litter functional structure (CWM and FDis) for each treatment was calculated based on initial litter traits (the concentrations of carbon (C), nitrogen (N), lignin, cellulose and initial C/N) of each species and its relative abundance. Specifically, we proposed the following hypotheses: First, non‐additive RMEs would be found common (Liu et al., 2016; Su et al., 2020); second, both evenness and dominant species identity would show significant effects on litter decomposition rate and remaining C/N (Li et al., 2013; Lin et al., 2013); and third, initial litter FDis would show a positive association with litter decomposition rate (mass loss rate, C loss rate and/or N loss rate), supporting Tilman's niche complementarity hypothesis (Tilman et al., 1997).

## 2 MATERIALS AND METHODS

### 2.1 Study materials

The litter of *L. chinensis*, *S. centauroides*, *A. capillaris* and 0–10 cm layer soil were obtained at Huihe National Field Scientific Observation and Research Station (118°04.8′–119°04.5′ E, 48°00.0′–48°05.7′ N) in Hulun Buir Steppe, China, and then were brought to Nankai University (117°17′ E, 39°10′ N). In the central area of Hulun Buir Steppe, *L. chinensis*, *S. centauroides* and *A. capillaris* co‐exist in most of natural communities, with their relative abundance varying across retrogressive successions. Air‐dried litter was homogenized and stored at room temperature for the following experiment. Before this experiment, five sub‐samples for each air‐dried litter were weighted (M_a_), and then were oven‐dried at 70°C to constant weight (M_c_). The M_c_/M_a_ for each litter was calculated as litter mass correction factor (f_m_).

### 2.2 Experimental design

We adopted a two‐factor experimental design using the litter of three species: *L. chinensis*, *S. centauroides* and/or *A. capillaris* (Table S1). The first factor included four types of evenness, namely high evenness, medium evenness, low evenness and single species. The value of evenness was calculated according to the evenness formula (Ricotta & Avena, 2003) based on the litter mass proportion of three species within each treatment. As a result, the evenness value of 1.5:1:1 was 0.982 (high evenness), that of 5:1:1 was 0.725 (medium evenness), that of 15:1:1was 0.404 for low evenness and that of single species was 0. The single‐species treatment was also used to calculate RMEs. The second factor was dominant species identity which was the species with the highest relative mass within litter mixture treatments; thus, the second factor included three species. We collected the remaining litter at three different decomposition times (30, 69 and 127 days of decomposition), with three replicates per treatment. In total, there were 108 litter samples (4 evenness × 3 dominant species × 3 collection times × 3 replicates).

At the beginning of the experiment, air‐dried litter which was converted into 1.19 g (initial mass, M_o_) of oven‐drying litter by formula M_a_ * f_m_ (M_a_: air‐dried litter; f_m_: litter mass correction factor) was put in a 1‐mm mesh nylon litterbag (10 cm × 15 cm), and then the litterbag was put in a plastic cultivation basin (12 cm in diameter) with 50 g adapted soil whose soil moisture content was (30 ± 2) %, close to the maximum water holding capacity. To optimize the environmental conditions, the experiment was performed in a climate incubator at a controlling temperature (25°C) with 24 h of darkness and (30 ± 2) % soil moisture content which was monitored by an ECH_2_O Check twice a week. The cultivation basins were placed randomly, and the position was changed once a week to avoid position effects. The experiment began on 11‐Nov‐2014, and collections of litter were performed on 11‐Dec‐2014 (30‐day decomposition), 18‐Jan‐2015 (69‐day decomposition) and 17‐Mar‐2015 (127‐day decomposition).

### 2.3 Trait measurement and calculation

#### 2.3.1 Initial litter characteristics

For litter of each species, each sub‐sample of oven‐drying litter were milled to powder for measurement of litter C and N concentrations (C_I‐concentration_ and N_I‐concentration_) by using an element analyser (Elementar, Hanau, Germany), and litter lignin and cellulose concentrations (lignin_I‐concentration_ and cellulose_I‐concentration_) by Van Soest method (Van Soest et al., 1991). The litter C or N content for each species in a certain treatment was the multiplication of the C_I‐concentration_ (or N_I‐concentration_) and its corresponding mass, and the sum of all species' content within the treatment was the initial C (or N) content (C_o_ or N_o_) for the treatment.

We quantified the litter character of each dominant species by sum approach with initial traits value (initial C, N, lignin and cellulose concentration, and C/N) by *z*‐score transformation. Considering a positive relationship between N concentration and decomposition rate, negative relationships between C, lignin, cellulose concentration and initial C/N and decomposition rate (Migliorini et al., 2018), the data of the last four traits were transformed by a negative mapping function *r*_*i*_(*f*_*i*_) = −*f*_*i*_. The character value was 5.54 for *L. chinensis* (high quality litter), 0.25 for *S. centauroides* (medium quality litter) and −5.80 for *A. capillaris* (low quality litter) (Table S3).

#### 2.3.2 Initial litter functional structure

Initial litter functional structure, FDis for all traits and CWM for each trait, was calculated based on the initial concentrations of C, N, lignin, cellulose and initial C/N and species relative mass percentage with packages ‘*psych*’ and ‘*FD*’ (Laliberte & Legendre, 2010) in R version 4.2.1. For the single‐species treatment, the FDis value was zero, and the CWM value was the trait average value of litter.

#### 2.3.3 Litter decomposition characteristics

For each collection (litterbag), litter was washed clean, oven‐dried to constant weight (remaining mass, M_R_) at 70°C and milled to powder for the measurement of remaining concentrations of C (C_R‐concentration_) and N (N_R‐concentration_) by using an element analyser (Elementar, Hanau, Germany). The content of remaining litter C or N (C_R_ or N_R_) was the multiplication of C_R‐concentration_ (or N_R‐concentration_) and its remaining mass (M_R_), and the remaining litter C/N was calculated as the ratio of C_R_ to N_R_.

For each collection, the observed litter mass loss rate, C loss rate and N loss rate were calculated by the following formulas respectively.

$$\text{Mass loss rate}=\left(M_{o}-M_{R}\right)/M_{0}\times 100\%.$$

$$C\;\text{loss rate}=\left(C_{o}-C_{R}\right)/C_{0}\times 100\%.$$

$$N\;\text{loss rate}=\left(N_{o}-N_{R}\right)/N_{0}\times 100\%.$$

#### 2.3.4 Relative mixture effect (RME) on mass loss

For a certain litter mixture treatment, the RME was calculated by the following formula (Wardle et al., 1997).

$$\mathrm{RME}=\left(\left({\text{Mass}}_{\text{Loss}-\mathrm{obs}}-{\text{Mass}}_{\text{Loss}-\exp}\right)/{\text{Mass}}_{\text{Loss}-\exp}\right)\times 100\%,$$

where Mass_Loss‐obs_ was the difference between M_0_ and M_R_, and Mass_Loss‐obs_ was calculated as the sum of multiplications between Mass_Loss‐obs_ of each litter under the single‐species treatment and its initial mass percentage in the mixture treatment.

### 2.4 Data analysis

All data used in this study met a normal distribution and homogeneity of variance.

First, one‐way ANOVA was used to analyse the litter characteristics difference among species in initial chemical composition content (C, N, lignin, cellulose) and C/N (Table S3).

Second, generalized linear models (GLMs) were used to analyse the effects of evenness, dominant species identity and their interactions on litter decomposition rate (mass loss rate, C loss rate, N loss rate), remaining litter C/N and RMEs on mass loss for each decomposition time in SPSS version 27.0 (IBM, USA). For the variable that was significantly affected by the interactions between evenness and dominant species identity, a simple‐effect analysis was used to evaluate the difference in their mean values among levels of one factor under a certain level of the other factor.

Third, the difference between RMEs and zero was estimated by one sample *t* tests in SPSS version 27.0. If the value is significantly lower than zero, it indicates an antagonistic effect (negative RME); if the value is significantly higher than zero, it indicates a synergistic effect (positive RME); and if the value is non‐significantly different from zero, it indicates an additive effect.

Fourth, Spearman correlation analysis was used for initial litter functional structure (FDis and CWM for each trait) and litter characteristics (mass loss rate, C loss rate, N loss rate and remaining litter C/N) with packages of ‘*Hmisc*’ and ‘*Performance Analytics*’ (Yadav & Roychoudhury, 2018) in R.

**FIGURE 1:**
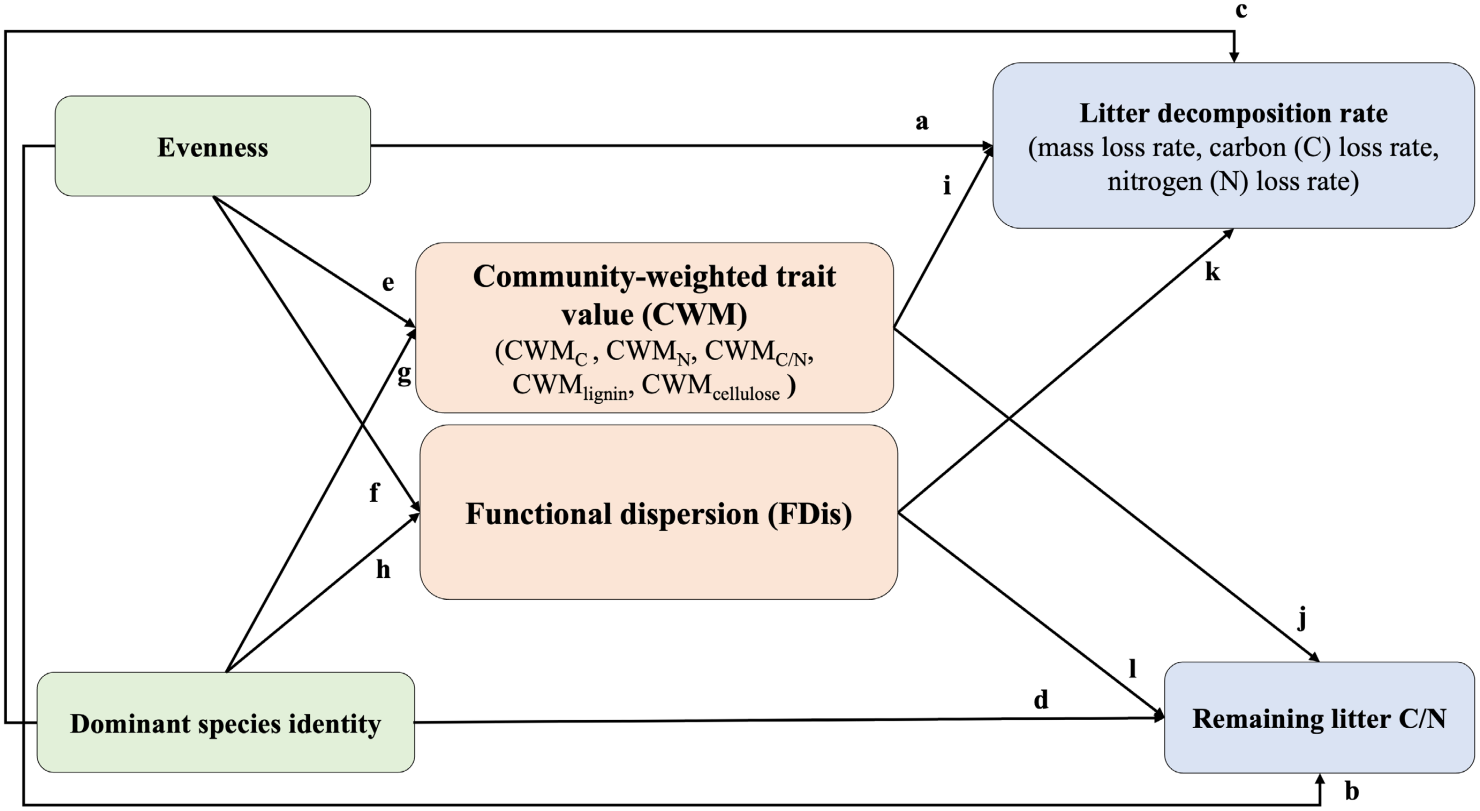
A priori model based on the existing system theory (Details in Appendix S1, Table S4).

Finally, to test the causal relationships between predictors and variables of litter decomposition processes (mass loss rate, C loss rate, N loss rate and remaining litter C/N), confirmatory path analyses were carried out using the package ‘*piecewis*eSEM’ (Lefcheck, 2016) in R, with all predictors as fixed factors, including evenness (0, 0.404, 0.725, 0.982), dominant species identity (−5.80, 0.25, 5.54), initial litter functional structure (FDis, CWM_C_, CWM_N_, CWM_C/N_, CWM_lignin_ and CWM_cellulose_) and plant composition as a random factor. Additionally, the AICc procedure was carried out to select the most appropriate predictors using the C statistic, and then the model with the lowest AIC value was selected as the best model if several models were not rejected. Before the analyses, a priori model (Figure 1) was constructed based on existing theories (Table S4).

## 3 RESULTS

### 3.1 Litter decomposition characteristics

**FIGURE 2:**
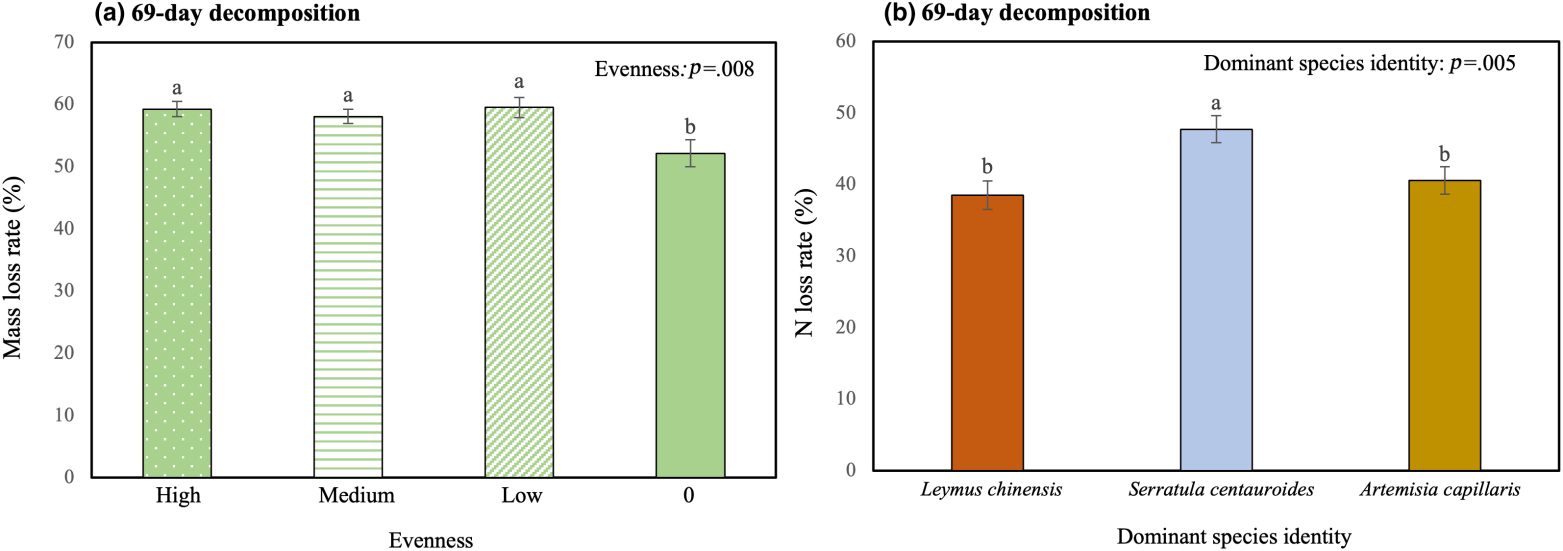
Effects of evenness (four shapes, high evenness: white dots; moderate evenness: horizontal lines; low evenness: white slashes; single decomposition: solid padding) and dominant species identity (three colours, *Leymus chinensis*: red; *Serratula centauroides*: blue; *Artemisia capillaris*: gold) on litter mass loss rate (a), litter N loss rate (b) after 69‐day decomposition. The columns with the same letters indicate non‐significant differences (*p* > .05) between or among these treatments.

After 69‐day decomposition, evenness significantly (*F*_3,32_ = 4.745, *p* < .05) affected mass loss rate (Table 1), with the value of single species being 6.79% lower than any of the other treatments (Figure 2a, Table S5). Dominant species identity significantly (*F*_2,33_ = 6.336, *p* < .05) affected N loss rate (Table 1), with treatment dominated by *S. centauroides* being at least 9.26% higher than that dominated by any of the other species (Figure 2b, Table S6).

**TABLE 1:** The effects of evenness, dominant species identity and their interactions on litter decomposition characteristics after 30‐, 60‐ and 127‐day decomposition by generalized linear models (GLMs).

Variable	Decomposition time	Evenness (df = 3)		Dominant species identity (df = 2)		Evenness × dominant species identity (df = 6)	
		*F*	*p*‐value	*F*	*p*‐value	*F*	*p*‐value
Mass loss rate	30 days	1.765	.181	0.472	.630	0.767	.603
	69 days	5.488	.005**	2.151	.138	1.451	.237
	127 days	0.315	.814	3.017	.068	0.936	.487
Carbon (C) loss rate	30 days	2.708	.068	1.171	.327	1.972	.110
	69 days	0.6	.621	0.387	.684	1.48	.227
	127 days	1.547	.228	0.764	.477	2.155	.084
Nitrogen (N) loss rate	30 days	1.375	.274	0.008	.992	0.405	.868
	69 days	1.06	.385	7.393	.003**	1.888	.124
	127 days	1.641	.206	1.429	.259	1.508	.218
Remaining litter C/N	30 days	0.497	.688	26.438	<.001***	4.864	.002**
	69 days	8.654	<.001***	63.926	<.001***	10.161	<.001***
	127 days	5.346	.006**	50.980	<.001***	7.453	<.001***

*Note*: Values in bold style indicate the significant effects of the factor (*p*‐value < .05). Meanwhile, ** and *** indicate that the effects of the factor are significant at .001 ≤ *p* < .01 and *p* < .001, respectively.

**FIGURE 3:**
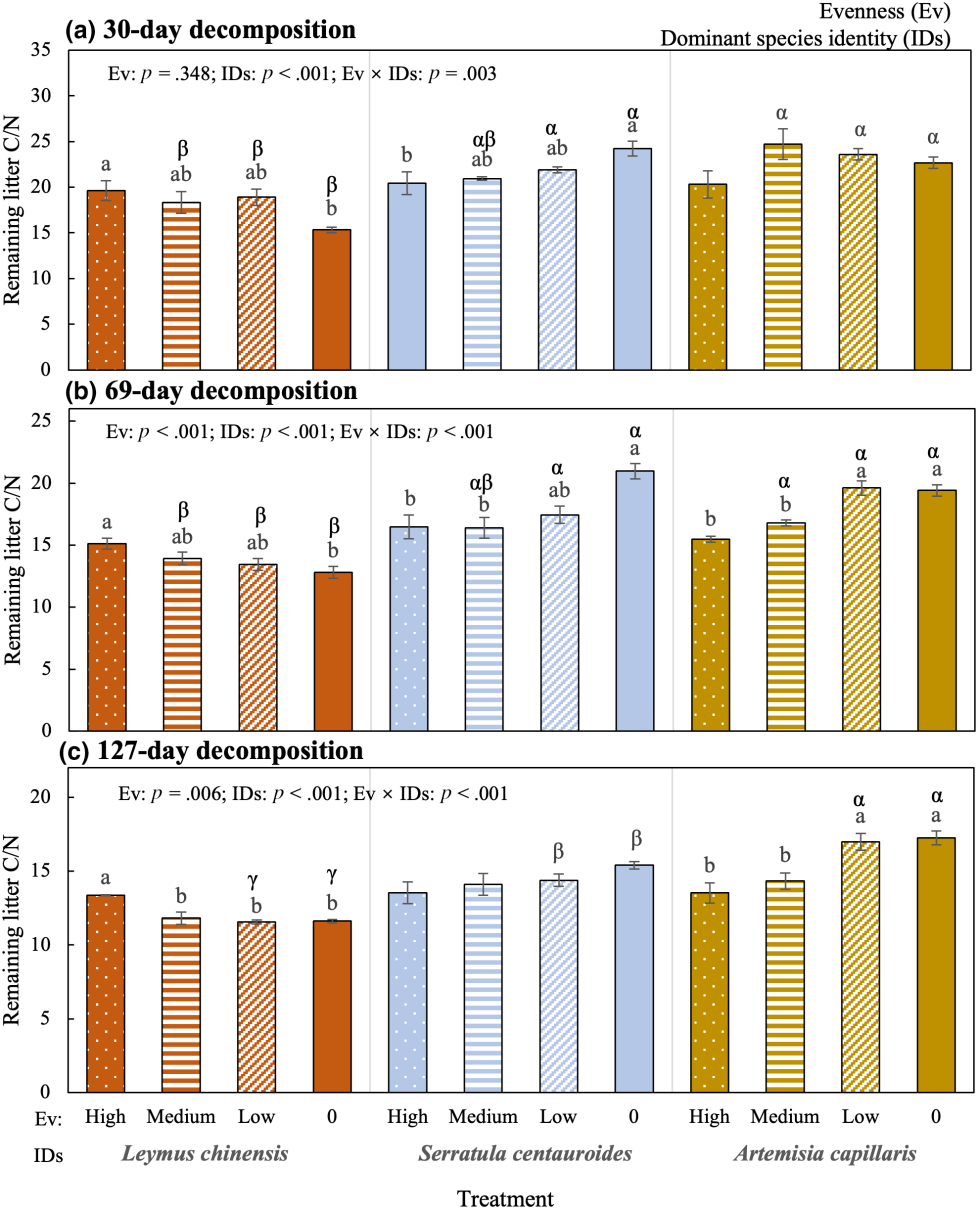
The effects of evenness (four shapes, high evenness: white dots; moderate evenness: horizontal lines; low evenness: white slashes; single decomposition: solid padding) and dominant species identity (three colours, *Leymus chinensis*: red; *Serratula centauroides*: blue; *Artemisia capillaris*: gold) on the remaining litter C/N after 30‐day decomposition (a), 69‐day decomposition (b) and 127‐day decomposition (c). The columns with the same English letters indicate non‐significant differences (*p* > .05) between or among these treatments under treatment dominated by a certain species, and those with the same Greek letters indicate non‐significant (*p* > .05) differences between or among the treatments dominated by different species under a certain evenness level.

The remaining C/N was significantly affected by evenness, dominant species identity and their interactions for all decomposition times except for the effect of evenness after 30‐day decomposition (Table 1), and the detailed results by simple effect analyses were shown in Figure 3. For treatments dominated by *L. chinensis*, the remaining C/N under high evenness treatment was higher than single‐species treatment after 30‐day and 69‐day decomposition and that of any of the other treatments after 127‐day decomposition. For treatments dominated by *S. centauroides*, the remaining C/N of single‐species treatment was higher than high evenness treatment after 30‐day decomposition and under the high or medium evenness treatment after 69‐day decomposition, while there was no significant difference among evenness treatments after 127‐day decomposition. For treatments dominated by *A. capillaris*, the remaining C/N under the single‐species treatment and low evenness treatment was higher than that under the medium or high evenness treatment after 69‐day and 127‐day decomposition, while there was no significant difference among evenness treatments after 30‐day decomposition.

For high evenness treatments, the differences among dominant species identity treatments were not significant for any of the three decomposition times. For medium evenness treatments, the remaining C/N under the treatment dominated by *A. capillaris* was higher than that under the treatment dominated by *L. chinensis*, and the treatment dominated by *S. centauroides* showed no significant difference from any of the other two treatments after 30‐day and 69‐day decomposition. There was no significant difference among the treatments dominated by different species. For low evenness treatment and single‐species treatment, the remaining C/N under the treatment dominated by *L. chinensis* was lower than that under any of the two treatments after 30‐day and 69‐day decomposition, and it was lower than treatment dominated by *S. centauroides* significantly, with the highest value under the treatment dominated by *A. capillaris* after 127‐day decomposition.

### 3.2 Relative mixture effects (RMEs)

**FIGURE 4:**
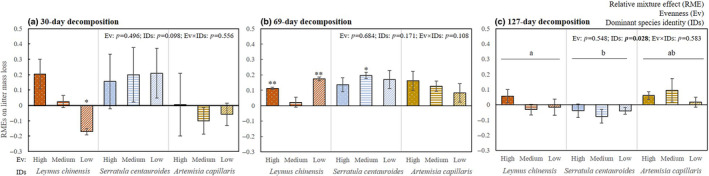
The effects of evenness (four shapes, high evenness: white dots; moderate evenness: horizontal lines; low evenness: white slashes; single decomposition: solid padding) and dominant species identity (three colours, *Leymus chinensis*: red; *Serratula centauroides*: blue; *Artemisia capillaris*: gold) on the relative mixture effects of litter mass loss after 30‐day decomposition (a), 69‐day decomposition (b) and 127‐day decomposition (c). The lines with the same letters indicate non‐significant differences (*p* > .05) between treatments dominated by different species. * and ** indicate that the differences between relative mixture effect and zero are significant at .01 ≤ *p* < .05 and .001 ≤ *p* < .01, respectively.

By single sample *t* tests, the value of RME was significantly lower than zero under the low evenness treatment dominated by *L. chinensis* after 30‐day decomposition (Figure 4a), suggesting the relatively lower litter mass loss than expected. In addition, the RME values were significantly higher than zero under the high and low evenness treatments dominated by *L. chinensis* and under the medium evenness treatment dominated by *S. centauroides* after 69‐day decomposition (Figure 4b), suggesting the relatively higher litter mass loss than expected.

By GLMs, dominant species identity had a significant effect on RMEs after 127‐day decomposition (*F*_5,30_ = 1015.86, *p* < .05), with the value under the treatment dominated by *S. centauroides* being lower than that under the treatment dominated by *L. chinensis* (Figure 4c).

### 3.3 Relationships between initial litter functional structure and litter decomposition characteristics

**FIGURE 5:**
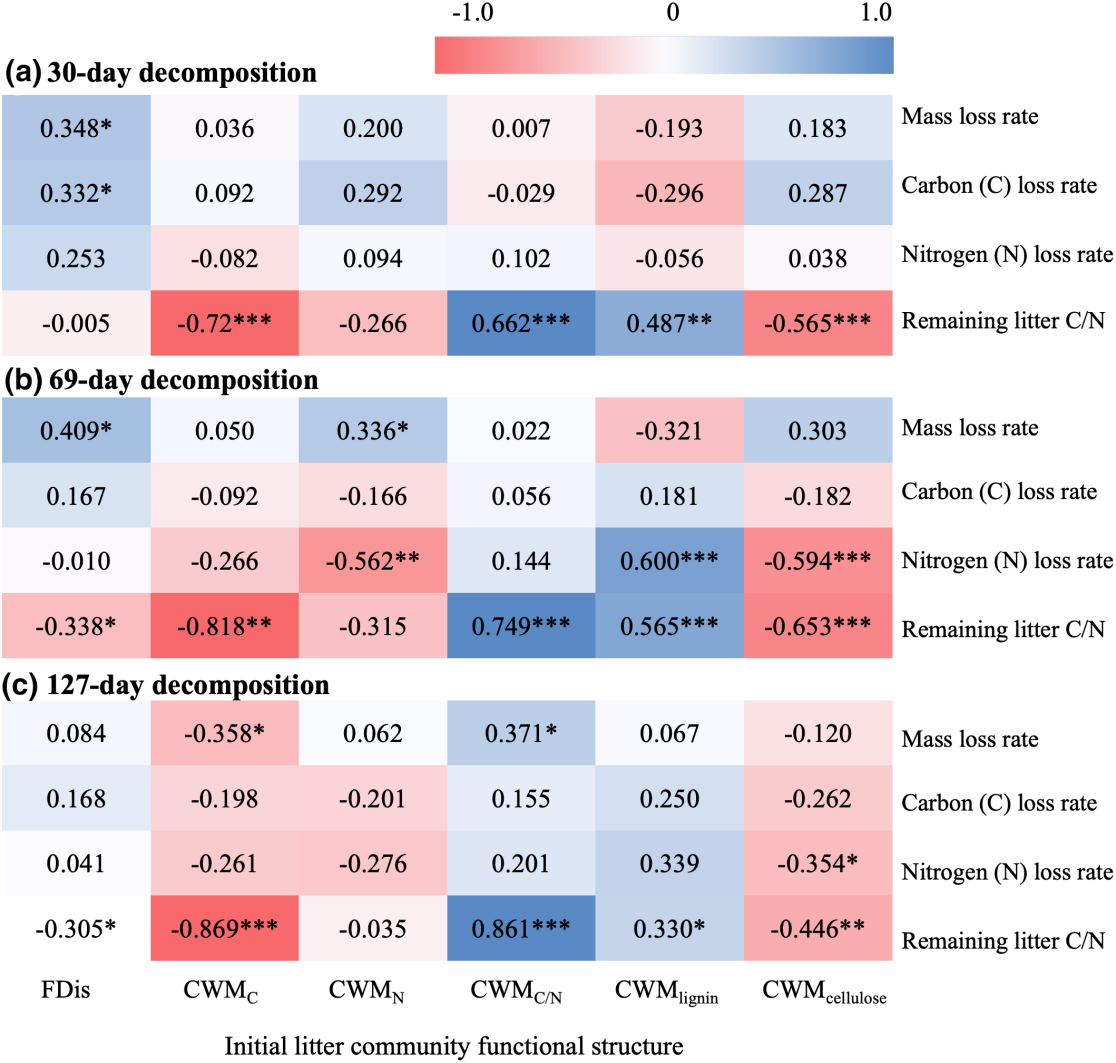
Spearman correlation coefficients and the fitting degree between initial litter functional structure and litter decomposition characteristics variables. The darkness of colour indicates the degree of correlation and the significant correlation is marked by * (.01 ≤ *p* < .05), ** (.001 ≤ *p* < .01) or *** (*p* < .001) respectively.

The mass loss rate was positively correlated with initial litter FDis after 30‐day and 69‐day decomposition, with initial litter CWM_N_ after 69‐day decomposition and with CWM_C/N_ after 127‐day decomposition, and was negatively correlated with initial litter CWM_C_ after 69‐day decomposition (Figure 5a–c). The C loss rate was positively correlated with initial litter FDis after 30‐day decomposition (Figure 5a). The N loss rate was negatively correlated with initial litter CWM_N_ after 69‐day decomposition, with initial litter CWM_cellulose_ after 69‐day and 127‐day decomposition, and was positively correlated with initial litter CWM_lignin_ after 69‐day decomposition (Figure 5b,c).

The remaining C/N showed a negative correlation with initial litter CWM_C_ and CWM_cellulose_ and a positive correlation with initial litter CWM_C/N_ and CWM_lignin_ for each decomposition time, and also a negative correlation with initial litter FDis after 69‐day decomposition (Figure 5b).

### 3.4 The direct and indirect effects of evenness and dominant species identity on litter decomposition characteristics

**FIGURE 6:**
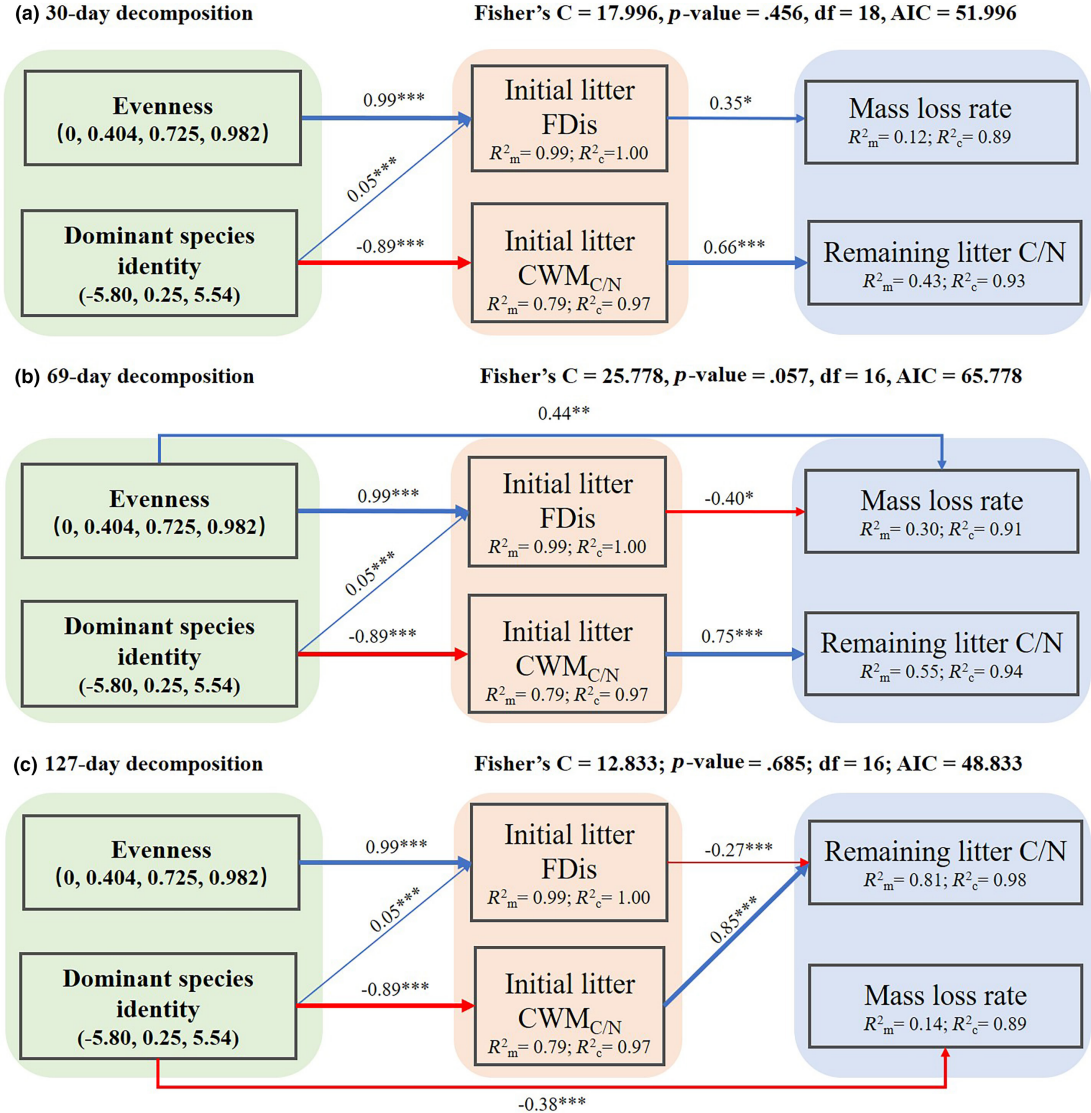
The causal relationship between species evenness, dominant species identity, initial litter functional structure (FDis and CWM) and litter decomposition characteristics after 30‐day decomposition (a), 69‐day decomposition (b) and 127‐day decomposition (c). The width of each arrow is proportional to the standardized path coefficients, with blue lines for positive correlations and red lines for negative correlations. *R* ^2^_m_: variance explained by fixed factor; *R* ^2^_c_: variance explained by both fixed and random factors. *, ** and *** indicate that the associations are significant at .01 ≤ *p* < .05, .001 ≤ *p* < .01 and *p* < .001 respectively.

By confirmatory path analyses, only two decomposition characteristics, mass loss rate and remaining C/N, were included in the final models which explained over 89% of the variance (Figure 6). Importantly, dominant species identity showed an indirect effect on remaining C/N for any of the three decomposition times, which was mediated by initial litter CWM_C/N_ (Figure 6). Specifically, after 69‐day decomposition, evenness showed a direct positive effect on mass loss rate; both evenness and dominant species identity showed indirect effects on mass loss rate, which was mediated by initial litter FDis (Figure 6b). After 127‐day decomposition, dominant species identity showed a direct negative effect on mass loss rate; both evenness and dominant species identity showed indirect effects on the remaining C/N, which was mediated by initial litter FDis (Figure 6c).

## 4 DISCUSSION

### 4.1 Interactions of species evenness and dominant species identity on litter decomposition characteristics

Any changes in evenness and dominant species identity would change litter resources and consequently microbial community characteristics, followed by plant community (Bonanomi et al., 2010; Zhang et al., 2020b), therefore, the significant interactive effect of evenness and dominant species identity on ecosystem functioning would be expected. For example, a study has found that one or more dominant species may have a significant effect on productivity of a grassland ecosystem in locations with low species evenness and that the increase or decrease of a species may have a significant effect on productivity in locations with high evenness (Smith & Knapp, 2003). Unfortunately, empirical proofs from litter decomposition experiments were very rare, Li et al. (2013) have found that it is dominant species identity but not evenness or the interactions between dominant species identity and evenness significantly affect litter mass loss rate. In the present study, only remaining C/N but not mass loss rate or nutrient loss rate was significantly affected by the interactive effect of evenness and dominant species identity (Figure 3). Besides, litter C/N is usually high in infertile soil regions, such as litter used in this study, which would cause decreasing in relative decomposition rate (Austin et al., 2014) and then would affect the estimation of effects of treatment factors. This study implies that litter decomposition or its response to different treatments is more complicated than it is expected. Therefore, we should pay more attention to this topic in the context of global change which might have led to occurring rapid community dynamics.

Although remaining litter C/N could not reflect litter decomposition rate in a direct way, it plays an important indicative role in litter decomposition processes because it can significantly regulate litter and soil microbial community (Li et al., 2021; Ye et al., 2019) and can regulate the release and fixation of total N in the decomposition processes (Pei et al., 2019). With the progresses of decomposition, the remaining litter C/N and the value difference between treatments would decrease (Ye et al., 2019), which would decrease the regulatory effect of litter C/N on litter decomposition. In natural tropical Panama, researchers have found that the litter decomposition processes of six native tree species are highly dependent on initial litter C/N and that decomposition rate slows down with increasing initial litter C/N (Scherer‐Lorenzen et al., 2007). Similar with the previous studies, the present study showed that initial litter CWM_C/N_ mediated positively the indirect effects of dominant species identity on remaining litter C/N (Figure 6). Furthermore, the values of remaining litter C/N are markedly dependent on the initial litter C/N of different species even though their variations with the progresses of decomposition were similar among different species (Abelho & Canhoto, 2020). In this study, after 127‐day decomposition, when the mass loss rate was higher than 50%, the remaining litter C/N was significantly affected by evenness, dominant species identity and their interactions. Both findings in this study support that initial litter C/N is a very important litter quality parameter that would show a long‐term and significant effect on litter decomposition processes (Talbot & Treseder, 2012), especially for the litter of dominant species.

### 4.2 Effect of dominant species identity on litter decomposition characteristics

The findings that dominant species identity significantly affected litter N loss rate after 69‐day decomposition, with treatment dominated by *S. centauroides* being at least 9.26% higher than that dominated by any of the other species and that dominant species litter quality was negatively associated with mass loss rate after 127‐day decomposition, partially support the second hypothesis in this study. Dominant species identity or plant functional group significantly affecting litter decomposition characteristics was found in previous studies (Bray et al., 2012; Yang et al., 2021). For example, Yang et al. (2021) have found that the litter removal of dominant species negatively affects litter decomposition of *Elymus nutans*. Li et al. (2013) found that the effects of leaf litter evenness on decomposition depend on which plant functional group is dominant. Dickson and Wilsey (2009) found that dominant species identity has a significant effect on litter mass loss. In the semi‐arid grasslands of Inner Mongolia, studies have shown that the decomposition rate is highest in litter mixtures dominated by dwarf shrubs and lowest in litter mixtures dominated by moss plants (Zhang et al., 2022). It has been demonstrated that chemical characteristics of both initial litter and decomposed litter show significant effects on microbial community composition and structure which would regulate specific litter decomposition processes by feedback (Fanin et al., 2014; Veen et al., 2021). These findings indicate that the changes in dominant species would change the nutrient cycle rate even in the same sites, which would be important for us to understand community succession and to give advice on practice of community restoration.

It is common that initial litter C/N has particularly strong positive effects on the remaining litter mass or negative effects on mass loss rate (Zhang et al., 2020a, 2022). By a 2‐year litter decomposition experiment using two tree species, *Castanopsis sieboldii* and *Schima wallichii* in a subtropical evergreen broad‐leaved forest in Okinawa, Japan, the researchers have shown a negative association between litter mass loss rate and initial litter C/N (Xu et al., 2004). However, Liu et al. (2012) noted that there would be a positive association between litter mass loss rate and initial litter C/N when initial litter C/N is higher than 20 which is specified as the critical level. In the present study, initial litter C/N was higher than the critical level (20) (Table S2), and the relationship between CWM_C/N_ and mass loss rate was positive after 127‐day decomposition (Figure 5), which provides empirical support for the opinion of Liu et al. (2012).

### 4.3 Effect of evenness on litter decomposition characteristics

The finding that evenness showed a positive effect on mass loss rate after 69‐day decomposition indicated that the increasing evenness would promote litter decomposition (Ward et al., 2010; Zhang et al., 2017). Initial litter FDis was positively associated with evenness, and it mediated the indirect effect of evenness on mass loss rate after 30‐day decomposition (Figure 6a); moreover, it showed significantly positive associations with mass loss rate, C loss rate after 30‐day decomposition. These results support that the niche complementarity hypothesis plays a role in regulating nutrient cycles (3rd hypothesis in this study). Litter mixture treatments in this study consisted of three species; thus, FDis variation among different mixture treatments was affected by SRA of litter. In a two‐year decomposition experiment carried out in the northern Greater Khingan Mountains in northeast China, researchers have found that FDis significantly increases with increasing evenness and that initial litter FDis shows a positive association with the mass loss rate (Zhang et al., 2020a). The microbial decomposition activity varying with different evenness treatments and relative abundance of dominant species of litter has been found in several studies (Ferreira et al., 2017; Urbanová et al., 2015). For example, Pereira and Ferreira (2022) have found that the microbial decomposition activity decreases as the proportion of *Acacia melanoxylon* increases. Additionally, a previous study has demonstrated that the litter decomposition rate of a relatively lower evenness is similar to that of its single dominant litter because the microbial community would tend to be simplified in the single or monodominant litter (Zhou et al., 2020). These findings demonstrate that a greater FDis would promote the activities of different microbial communities because of the higher resource availability (Valencia et al., 2018; Zuo et al., 2016).

### 4.4 Relative mixture effect

In the present study, we have not found a significant effect of evenness on RMEs. Similar result has been reported in a previous study, King et al. (2002) found that evenness has little effect on litter RMEs. The majority of RMEs (23/27) were additive (Figure 4), which did not support the first hypothesis in this study and was inconsistent with other studies (Liu et al., 2016; Pires et al., 2018). In detail, only three significantly positive RMEs and one significantly negative RME were found within 27 treatments, supporting that additive effects dominate RMEs of mixing litter with different evenness degrees in arid and semi‐arid climatic conditions (Canessa et al., 2022). After 127‐day decomposition, there was no non‐additive RMEs, which suggested that the RMEs gradually approached zero with progress of decomposition (Cassart et al., 2020; Su et al., 2023).

There were non‐additive RMEs under the treatments dominated by *L. chinensis* (Figure 4), suggesting that the abundance changes of *L. chinensis* in litter mixtures would affect litter decomposition by non‐additive effect. By GLMs, we found that only dominant species identity showed a significant effect on RMEs on mass loss after 129‐day decomposition, suggesting that the responses of litter mass loss in mixtures are more dependent on litter qualities and decomposition stages, but not evenness (Handa et al., 2014). In both forests and grassland communities, dominant species identity affecting RMEs has been reported (Li et al., 2013; Zhang et al., 2016). These findings suggest the significance of unique litter decomposition identities in the litter mixture (Zhou et al., 2020), and also show that the changes of dominant species identity would lead to synergism or antagonism (non‐additive RMEs) in multispecies litter mixtures (Butenschoen et al., 2014; Handa et al., 2014).

## 5 CONCLUSION

This study provides empirical support that litter species evenness and dominant species identity interactively affect litter remaining C/N but not mass (C, N) loss rate. Besides, initial litter C/N mediated the indirect effect of dominant species identity on remaining litter C/N. Initial litter FDis mediated the indirect effects of both evenness and dominant species identity on mass loss rate after 30‐ and 69‐day decomposition and on remaining C/N after 127‐day decomposition. Considering the feedback between community composition and structures and litter decomposition, that is, community composition and evenness are able to influence litter composition and evenness which in turn can affect community conditions, this study not only provides insights into BEF mechanisms, especially for communities during retrogressive succession in semi‐arid grasslands in the context of global changes, but also helps to understand community succession processes and the ecological consequences.

## AUTHOR CONTRIBUTIONS

**Baijie Fan:** Resources (equal); visualization (equal); writing – original draft (equal); writing – review and editing (equal). **Ziqing Gong:** Methodology (equal); software (equal); writing – original draft (equal); writing – review and editing (equal). **Xiaojing Xin:** Investigation (equal); resources (equal); writing – original draft (equal); writing – review and editing (equal). **Yulin Liu:** Project administration (equal); software (equal); writing – review and editing (equal). **Luoyang He:** Formal analysis (equal); software (equal). **Yubao Gao:** Data curation (equal); writing – review and editing (equal). **Anzhi Ren:** Methodology (equal); validation (equal); writing – review and editing (equal). **Nianxi Zhao:** Funding acquisition (equal); writing – review and editing (equal).

## CONFLICT OF INTEREST STATEMENT

The authors declare that they have no conflict of interest. This article does not contain any studies with human participants or animals performed by any of the authors.

## Supporting information


Appendix S1.

## Data Availability

The data that support the findings of this study will be available in Dryad at https://datadryad.org/stash/share/wAdt1r0OJ672t‐gEStamkvTq9G6I3jahY464DrCSlg8.

## References

[ece311052-bib-0001] Abelho, M. , & Canhoto, C. (2020). The role of carbon, nitrogen, and phosphorus in leaf decomposition mediated by aquatic fungi. Limnetica, 39, 275–282. 10.23818/limn.39.18

[ece311052-bib-0002] Austin, A. T. , Vivanco, L. , Gonzalez‐Arzac, A. , & Perez, L. I. (2014). There's no place like home? An exploration of the mechanisms behind plant litter‐decomposer affinity in terrestrial ecosystems. New Phytologist, 204, 307–314. 10.1111/nph.12959 25103145

[ece311052-bib-0003] Bonanomi, G. , Incerti, G. , Antignani, V. , Capodilupo, M. , & Mazzoleni, S. (2010). Decomposition and nutrient dynamics in mixed litter of Mediterranean species. Plant and Soil, 331, 481–496. 10.1007/s11104-009-0269-6

[ece311052-bib-0004] Bradford, M. A. , Berg, B. , Maynard, D. S. , Wieder, W. R. , & Wood, S. A. (2016). Understanding the dominant controls on litter decomposition. Journal of Ecology, 104, 229–238. 10.1111/1365-2745.12507

[ece311052-bib-0005] Bray, S. R. , Kitajima, K. , & Mack, M. C. (2012). Temporal dynamics of microbial communities on decomposing leaf litter of 10 plant species in relation to decomposition rate. Soil Biology & Biochemistry, 49, 30–37. 10.1016/j.soilbio.2012.02.009

[ece311052-bib-0006] Butenschoen, O. , Krashevska, V. , Maraun, M. , Marian, F. , Sandmann, D. , & Scheu, S. (2014). Litter mixture effects on decomposition in tropical montane rainforests vary strongly with time and turn negative at later stages of decay. Soil Biology & Biochemistry, 77, 121–128. 10.1016/j.soilbio.2014.06.019

[ece311052-bib-0007] Canessa, R. , van den Brink, L. , Berdugo, M. B. , Hattenschwiler, S. , Rios, R. S. , Saldana, A. , Tielbörger, K. , & Bader, M. Y. (2022). Trait functional diversity explains mixture effects on litter decomposition at the arid end of a climate gradient. Journal of Ecology, 110, 2219–2231. 10.1111/1365-2745.13946

[ece311052-bib-0008] Cassart, B. , Basia, A. A. , Jonard, M. , & Ponette, Q. (2020). Average leaf litter quality drives the decomposition of single‐species, mixed‐species and transplanted leaf litters for two contrasting tropical forest types in The Congo Basin (DRC). Annals of Forest Science, 77, 33. 10.1007/s13595-020-00942-4

[ece311052-bib-0009] Chen, Y. C. , Ma, S. Q. , Jiang, H. M. , Yangzom, D. , Cheng, G. W. , & Lu, X. Y. (2021). Decomposition time, chemical traits and climatic factors determine litter‐mixing effects on decomposition in an alpine steppe ecosystem in northern Tibet. Plant and Soil, 459, 23–35. 10.1007/s11104-019-04131-9

[ece311052-bib-0010] Cheng, Y. X. , Kamijo, T. , Tsubo, M. , & Nakamura, T. (2013). Phytosociology of Hulunbeier grassland vegetation in Inner Mongolia, China. Phytocoenologia, 43, 41–51. 10.1127/0340-269x/2013/0043-0540

[ece311052-bib-0011] Dangles, O. , & Malmqvist, B. (2004). Species richness‐decomposition relationships depend on species dominance. Ecology Letters, 7, 395–402. 10.1111/j.1461-0248.2004.00591.x

[ece311052-bib-0012] Diaz, S. , Lavorel, S. , de Bello, F. , Quetier, F. , Grigulis, K. , & Robson, M. (2007). Incorporating plant functional diversity effects in ecosystem service assessments. Proceedings of the National Academy of Sciences of the United States of America, 104, 20684–20689. 10.1073/pnas.0704716104 18093933 PMC2410063

[ece311052-bib-0013] Dickson, T. L. , & Wilsey, B. J. (2009). Biodiversity and tallgrass prairie decomposition: The relative importance of species identity, evenness, richness, and microtopography. Plant Ecology, 201, 639–649. 10.1007/s11258-008-9567-y

[ece311052-bib-0014] Fanin, N. , Hattenschwiler, S. , & Fromin, N. (2014). Litter fingerprint on microbial biomass, activity, and community structure in the underlying soil. Plant and Soil, 379, 79–91. 10.1007/s11104-014-2051-7

[ece311052-bib-0015] Ferreira, V. , Faustino, H. , Raposeiro, P. M. , & Gonçalves, V. (2017). Replacement of native forests by conifer plantations affects fungal decomposer community structure but not litter decomposition in Atlantic Island streams. Forest Ecology and Management, 389, 323–330. 10.1016/j.foreco.2017.01.004

[ece311052-bib-0016] Grime, J. P. (1998). Benefits of plant diversity to ecosystems: Immediate, filter and founder effects. Journal of Ecology, 86, 902–910. 10.1046/j.1365-2745.1998.00306.x

[ece311052-bib-0017] Guo, C. , Cornelissen, J. H. C. , Tuo, B. , Ci, H. , & Yan, E. R. (2020). Nonnegligible contribution of subordinates in community‐level litter decomposition: Deciduous trees in an evergreen world. Journal of Ecology, 108, 1713–1724. 10.1111/1365-2745.13341

[ece311052-bib-0018] Handa, I. T. , Aerts, R. , Berendse, F. , Berg, M. P. , Bruder, A. , Butenschoen, O. , Chauvet, E. , Gessner, M. O. , Jabiol, J. , Makkonen, M. , McKie, B. G. , Malmqvist, B. , Peeters, E. T. H. M. , Scheu, S. , Schmid, B. , van Ruijven, J. , Vos, V. C. A. , & Hättenschwiler, S. (2014). Consequences of biodiversity loss for litter decomposition across biomes. Nature, 509, 218–221. 10.1038/nature13247 24805346

[ece311052-bib-0070] Hubbell, S. P. , Ahumada, J. A. , Condit, R. , & Foster, R. B. (2001). Local neighborhood effects on long‐term survival of individual trees in a neotropical forest. Ecological Research, 16, 859–875. 10.1046/j.1440-1703.2001.00445.x

[ece311052-bib-0019] Jiang, G. S. , Liu, J. , Xu, L. , Yu, G. R. , He, H. L. , & Zhang, Z. B. (2013). Climate warming increases biodiversity of small rodents by favoring rare or less abundant species in a grassland ecosystem. Integrative Zoology, 8, 162–174. 10.1111/1749-4877.12027 23731812

[ece311052-bib-0020] Jiang, M. , He, L. Y. , Fan, B. J. , Wang, T. , Yang, N. , Liu, Y. L. , Xu, Y. , Dong, K. , Hao, G. , Chen, L. , Ren, A. , Zhao, N. , Wang, J. , & Gao, Y. (2022). Intraspecific more than interspecific diversity plays an important role on Inner Mongolia grassland ecosystem functions: A microcosm experiment. Science of the Total Environment, 826, 154134. 10.1016/j.scitotenv.2022.154134 35219658

[ece311052-bib-0021] King, R. F. , Dromph, K. M. , & Bardgett, R. D. (2002). Changes in species evenness of litter have no effect on decomposition processes. Soil Biology & Biochemistry, 34, 1959–1963. 10.1016/S0038-0717(02)00204-3

[ece311052-bib-0022] Laliberte, E. , & Legendre, P. (2010). A distance‐based framework for measuring functional diversity from multiple traits. Ecology, 91, 299–305. 10.1890/08-2244.1 20380219

[ece311052-bib-0023] Lefcheck, J. S. (2016). PIECEWISESEM: Piecewise structural equation modelling in R for ecology, evolution, and systematics. Methods in Ecology and Evolution, 7, 573–579. 10.1111/2041-210x.12512

[ece311052-bib-0024] Li, D. J. , Peng, S. L. , & Chen, B. M. (2013). The effects of leaf litter evenness on decomposition depend on which plant functional group is dominant. Plant and Soil, 365, 255–266. 10.1007/s11104-012-1337-x

[ece311052-bib-0025] Li, L. , Wen, Z. F. , Wei, S. G. , Lian, J. Y. , & Ye, W. H. (2022). Functional diversity and its influencing factors in a subtropical forest community in China. Forests, 13, 966. 10.3390/f13070966

[ece311052-bib-0026] Li, R. S. , Zhang, Y. Z. , Yu, D. , Wang, Y. , Zhao, X. X. , Zhang, R. H. , Zhang, W. , Wang, Q. , Xu, M. , Chen, L. , Wang, S. , Han, J. , & Yang, Q. (2021). The decomposition of green leaf litter is less temperature sensitive than that of senescent leaf litter: An incubation study. Geoderma, 381, 114691. 10.1016/j.geoderma.2020.114691

[ece311052-bib-0027] Lin, G. G. , Mao, R. , Zhao, L. , & Zeng, D. H. (2013). Litter decomposition of a pine plantation is affected by species evenness and soil nitrogen availability. Plant and Soil, 373, 649–657. 10.1007/s11104-013-1832-8

[ece311052-bib-0028] Liski, J. , Nissinen, A. , Erhard, M. , & Taskinen, O. (2003). Climatic effects on litter decomposition from arctic tundra to tropical rainforest. Global Change Biology, 9, 575–584. 10.1046/j.1365-2486.2003.00605.x

[ece311052-bib-0029] Liu, C. C. , Liu, Y. G. , Guo, K. , Zhao, H. W. , Qia, X. G. , Wang, S. J. , Zhang, L. , & Cai, X. (2016). Mixing litter from deciduous and evergreen trees enhances decomposition in a subtropical karst forest in southwestern China. Soil Biology & Biochemistry, 101, 44–54. 10.1016/j.soilbio.2016.07.004

[ece311052-bib-0030] Liu, J. , Wu, J. G. , Liu, F. Q. , & Han, X. G. (2012). Quantitative assessment of bioenergy from crop stalk resources in Inner Mongolia, China. Applied Energy, 93, 305–318. 10.1016/j.apenergy.2011.12.059

[ece311052-bib-0031] Loreau, M. , & Hector, A. (2001). Partitioning selection and complementarity in biodiversity experiments. Nature, 412, 72–76. 10.1038/3583573 11452308

[ece311052-bib-0032] McLaren, J. R. , & Turkington, R. (2011). Plant identity influences decomposition through more than one mechanism. PLoS One, 6, e23702. 10.1371/journal.pone.0023702 21858210 PMC3156744

[ece311052-bib-0033] Migliorini, G. H. , Srivastava, D. S. , & Romero, G. Q. (2018). Leaf litter traits drive community structure and functioning in a natural aquatic microcosm. Freshwater Biology, 63, 341–352. 10.1111/fwb.13072

[ece311052-bib-0034] Pei, G. T. , Liu, J. , Peng, B. , Gao, D. C. , Wang, C. , Dai, W. W. , Jiang, P. , & Bai, E. (2019). Nitrogen, lignin, C/N as important regulators of gross nitrogen release and immobilization during litter decomposition in a temperate forest ecosystem. Forest Ecology and Management, 440, 61–69. 10.1016/j.foreco.2019.03.001

[ece311052-bib-0035] Pereira, A. , & Ferreira, V. (2022). Increasing inputs of invasive N‐fixing acacia litter decrease litter decomposition and associated microbial activity in streams. Freshwater Biology, 67, 292–308. 10.1111/fwb.13841

[ece311052-bib-0036] Pires, A. P. F. , Srivastava, D. S. , Marino, N. A. C. , MacDonald, A. A. M. , Figueiredo‐Barros, M. P. , & Farjalla, V. F. (2018). Interactive effects of climate change and biodiversity loss on ecosystem functioning. Ecology, 99, 1203–1213. 10.1002/ecy.2202 29714828

[ece311052-bib-0037] Ricotta, C. , & Avena, G. (2003). On the relationship between Pielou's evenness and landscape dominance within the context of Hill's diversity profiles. Ecological Indicators, 2, 361–365. 10.1016/S1470-160x(03)00005-0

[ece311052-bib-0038] Scherer‐Lorenzen, M. , Bonilla, J. L. , & Potvin, C. (2007). Tree species richness affects litter production and decomposition rates in a tropical biodiversity experiment. Oikos, 116, 2108–2124. 10.1111/j.2007.0030-1299.16065.x

[ece311052-bib-0039] Setiawan, N. N. , Vanhellemont, M. , De Schrijver, A. , Schelfhout, S. , Baeten, L. , & Verheyen, K. (2016). Mixing effects on litter decomposition rates in a young tree diversity experiment. Acta Oecologica‐International Journal of Ecology, 70, 79–86. 10.1016/j.actao.2015.12.003

[ece311052-bib-0040] Smith, M. D. , & Knapp, A. K. (2003). Dominant species maintain ecosystem function with non‐random species loss. Ecology Letters, 6, 509–517. 10.1046/j.1461-0248.2003.00454.x

[ece311052-bib-0041] Su, J. S. , Zhao, Y. J. , & Bai, Y. F. (2023). Asymmetric responses of leaf litter decomposition to precipitation changes in global terrestrial ecosystem. Journal of Cleaner Production, 387, 135898. 10.1016/j.jclepro.2023.135898

[ece311052-bib-0042] Su, Y. J. , Cao, Y. H. , Ding, C. H. , Sun, Y. Y. , Cheng, K. L. , Zeng, R. S. , Song, Y. Y. , & Li, Z. F. (2020). Nonadditive allelopathic effects of decomposing mixed litters of *Eucalyptus urophylla* and *Acacia mangium* on radish, lettuce and *Paspalum notatum* . Allelopathy Journal, 49, 189–200. 10.26651/allelo.j/2020-49-2-1264

[ece311052-bib-0043] Swan, C. M. , Gluth, M. A. , & Horne, C. L. (2009). Leaf litter species evenness influences nonadditive breakdown in a headwater stream. Ecology, 90, 1650–1658. 10.1890/08-0329.1 19569379

[ece311052-bib-0044] Talbot, J. M. , & Treseder, K. K. (2012). Interactions among lignin, cellulose, and nitrogen drive litter chemistry‐decay relationships. Ecology, 93, 345–354. 10.1890/11-0843.1 22624316

[ece311052-bib-0045] Taylor, B. R. , Mallaley, C. , & Cairns, J. F. (2007). Limited evidence that mixing leaf litter accelerates decomposition or increases diversity of decomposers in streams of eastern Canada. Hydrobiologia, 592, 405–422. 10.1007/s10750-007-0778-3

[ece311052-bib-0046] Tilman, D. , Knops, J. , Wedin, D. , Reich, P. , Ritchie, M. , & Siemann, E. (1997). The influence of functional diversity and composition on ecosystem processes. Science, 277, 1300–1302. 10.1126/science.277.5330.1300

[ece311052-bib-0069] Tilman, D. , Reich, P. B. , Knops, J. , Wedin, D. , Mielke, T. , & Lehman, C. (2001). Diversity and productivity in a long‐term grassland experiment. Science, 294, 843–845. 10.1126/science.1060391 11679667

[ece311052-bib-0047] Tobner, C. M. , Paquette, A. , Gravel, D. , Reich, P. B. , Williams, L. J. , & Messier, C. (2016). Functional identity is the main driver of diversity effects in young tree communities. Ecology Letters, 19, 638–647. 10.1111/ele.12600 27072428

[ece311052-bib-0048] Urbanová, M. , Snajdr, J. , & Baldrian, P. (2015). Composition of fungal and bacterial communities in forest litter and soil is largely determined by dominant trees. Soil Biology & Biochemistry, 84, 53–64. 10.1016/j.soilbio.2015.02.011

[ece311052-bib-0049] Valencia, E. , Gross, N. , Quero, J. L. , Carmona, C. P. , Ochoa, V. , Gozalo, B. , Delgado‐Baquerizo, M. , Dumack, K. , Hamonts, K. , Singh, B. K. , Bonkowski, M. , & Maestre, F. T. (2018). Cascading effects from plants to soil microorganisms explain how plant species richness and simulated climate change affect soil multifunctionality. Global Change Biology, 24, 5642–5654. 10.1111/gcb.14440 30239067

[ece311052-bib-0050] Van Soest, P. J. , Robertson, J. B. , & Lewis, B. A. (1991). Methods for dietary fiber, neutral detergent fiber, and nonstarch polysaccharides in relation to animal nutrition. Journal of Dairy Science, 74, 3583–3597. 10.3168/jds.S0022-0302(91)78551-2 1660498

[ece311052-bib-0051] Veen, G. F. , ten Hooven, F. C. , Weser, C. , & Hannula, S. E. (2021). Steering the soil microbiome by repeated litter addition. Journal of Ecology, 109, 2499–2513. 10.1111/1365-2745.13662

[ece311052-bib-0052] Ward, S. E. , Ostle, N. J. , McNamara, N. P. , & Bardgett, R. D. (2010). Litter evenness influences short‐term peatland decomposition processes. Oecologia, 164, 511–520. 10.1007/s00442-010-1636-y 20431923

[ece311052-bib-0053] Wardle, D. A. (2013). Communities and ecosystems: Linking the aboveground and belowground components (Vol. 34, p. i). Princeton University Press.

[ece311052-bib-0054] Wardle, D. A. , Bonner, K. I. , & Nicholson, K. S. (1997). Biodiversity and plant litter: Experimental evidence which does not support the view that enhanced species richness improves ecosystem function. Oikos, 79, 247–258. 10.2307/3546010

[ece311052-bib-0055] Xu, L. , Gao, Q. , & Wang, Y. L. (2014). Species richness within a six‐year slope exclosure in a temperate grassland and its relationship with aboveground biomass. Ecology and Environmental Sciences, 23, 398–405. 10.16258/j.cnki.1674-5906.2014.03.017

[ece311052-bib-0056] Xu, X. N. , Hirata, E. , Enoki, T. , & Tokashiki, Y. (2004). Leaf litter decomposition and nutrient dynamics in a subtropical forest after typhoon disturbance. Plant Ecology, 173, 161–170. 10.1023/B:Vege.0000029319.05980.70

[ece311052-bib-0057] Yadav, M. L. , & Roychoudhury, B. (2018). Handling missing values: A study of popular imputation packages in R. Knowledge‐Based Systems, 160, 104–118. 10.1016/j.knosys.2018.06.012

[ece311052-bib-0058] Yang, X. D. , Yang, Z. , Warren, M. W. , & Chen, J. (2012). Mechanical fragmentation enhances the contribution of collembola to leaf litter decomposition. European Journal of Soil Biology, 53, 23–31. 10.1016/j.ejsobi.2012.07.006

[ece311052-bib-0059] Yang, X. L. , Wang, X. T. , Xiao, S. , Liu, Z. Y. , Zhou, X. H. , Du, G. Z. , Liu, K. , Wang, Y. , Chen, S. , & Nielsen, U. N. (2021). Dominant plants affect litter decomposition mainly through modifications of the soil microbial community. Soil Biology & Biochemistry, 161, 108399. 10.1016/j.soilbio.2021.108399

[ece311052-bib-0060] Ye, X. M. , Zhang, Y. , Chen, F. S. , Wang, G. G. , Fang, X. M. , Lin, X. F. , Wan, S. Z. , & He, P. (2019). The effects of simulated deposited nitrogen on nutrient dynamics in decomposing litters across a wide quality spectrum using a using a ^15^N tracing technique. Plant and Soil, 442, 141–156. 10.1007/s11104-019-04158-y

[ece311052-bib-0061] Zeng, W. X. , Xiang, W. H. , Fang, J. P. , Zhou, B. , Ouyang, S. , Zeng, Y. L. , Chen, L. , Lei, P. , Milcu, A. , & Valverde‐Barrantes, O. J. (2020). Species richness and functional‐trait effects on fine root biomass along a subtropical tree diversity gradient. Plant and Soil, 446, 515–527. 10.1007/s11104-019-04369-3

[ece311052-bib-0062] Zhang, W. D. , Chao, L. , Yang, Q. P. , Wang, Q. K. , Fang, Y. T. , & Wang, S. L. (2016). Litter quality mediated nitrogen effect on plant litter decomposition regardless of soil fauna presence. Ecology, 97, 2834–2843. 10.1002/ecy.1515 27859104

[ece311052-bib-0063] Zhang, X. H. , Sun, X. X. , & Mao, R. (2017). Effects of litter evenness, nitrogen enrichment and temperature on short‐term litter decomposition in freshwater marshes of Northeast China. Wetlands, 37, 145–152. 10.1007/s13157-016-0855-3

[ece311052-bib-0064] Zhang, X. H. , Wang, L. , Jiang, W. , & Mao, R. (2020a). Functional identity and functional diversity coregulate litter mixture decomposition and nitrogen release in boreal riparian forest ponds. Biogeochemistry, 151, 99–111. 10.1007/s10533-020-00716-0

[ece311052-bib-0065] Zhang, X. H. , Wang, Y. P. , Jiang, W. , & Mao, R. (2020b). Effect of expanded shrub litter on decomposition of graminoid litter in a temperate freshwater marsh. Plant and Soil, 451, 409–418. 10.1007/s11104-020-04536-x

[ece311052-bib-0066] Zhang, X. H. , Zhang, Y. H. , Jiang, S. S. , Song, C. C. , Zhang, J. B. , & Mao, R. (2022). Dominant species and evenness level coregulate litter mixture decomposition in a boreal peatland. Plant and Soil, 474, 423–436. 10.1007/s11104-022-05346-z

[ece311052-bib-0067] Zhou, S. X. , Butenschoen, O. , Barantal, S. , Handa, I. T. , Makkonen, M. , Vos, V. , Aerts, R. , Berg, M. P. , McKie, B. , van Ruijven, J. , Hättenschwiler, S. , & Scheu, S. (2020). Decomposition of leaf litter mixtures across biomes: The role of litter identity, diversity and soil fauna. Journal of Ecology, 108, 2283–2297. 10.1111/1365-2745.13452

[ece311052-bib-0068] Zuo, X. A. , Zhou, X. , Lv, P. , Zhao, X. Y. , Zhang, J. , Wang, S. K. , & Yue, X. (2016). Testing associations of plant functional diversity with carbon and nitrogen storage along a restoration gradient of Sandy grassland. Frontiers in Plant Science, 7, 189. 10.3389/fpls.2016.00189 26925089 PMC4759253

