# Supplementary material for: Both evenness and dominant species identity have effects on litter decomposition
Source: Ecol Evol. 2024 Feb 26;14(2):e11052. doi: 10.1002/ece3.11052 (PMC10896676; doi:10.1002/ece3.11052)
Supplement: Supplementary file 1 — Appendix S1. [file ECE3-14-e11052-s001.docx]

**Appendix A**

**Extra information on methods and results**

**belongs to the study:**

**Both evenness and dominant species identity have effects on litter decomposition**

**Baijie Fan, Ziqing Gong, Xiaojing Xin, Yulin Liu, Luoyang He, Yubao Gao, Anzhi Ren, Nianxi Zhao**

**Table S1** Experimental design.

| Group code | Litter species richness | Litter species evenness | Degree of evenness | Litter assemblages (g/litterbag) | | | Litter dominant species identity |
| --- | --- | --- | --- | --- | --- | --- | --- |
|  |  |  |  | *Leymus chinensis* | *Artemisia capillaris* | *Serratula centauroides* |  |
| 1 | 1 | 0 | 0 | 1.1901 | 0 | 0 | *L． chinensis* |
| 2 | 1 | 0 | 0 | 0 | 1.1905 | 0 | *A．capillaris* |
| 3 | 1 | 0 | 0 | 0 | 0 | 1.1903 | *S． centauroides* |
| 4 | 3 | 0.98 | High | 0.5110 | 0.3406 | 0.3409 | *L． chinensis* |
| 5 | 3 | 0.98 | High | 0.3400 | 0.5100 | 0.3406 | *A．capillaris* |
| 6 | 3 | 0.98 | High | 0.3406 | 0.3405 | 0.5107 | *S． centauroides* |
| 7 | 3 | 0.72 | Medium | 0.8504 | 0.1701 | 0.1705 | *L． chinensis* |
| 8 | 3 | 0.72 | Medium | 0.1704 | 0.8510 | 0.1710 | *A．capillaris* |
| 9 | 3 | 0.72 | Medium | 0.1702 | 0.1703 | 0.8507 | *S． centauroides* |
| 10 | 3 | 0.40 | Low | 1.0508 | 0.0707 | 0.0704 | *L． chinensis* |
| 11 | 3 | 0.40 | Low | 0.0703 | 1.0506 | 0.0705 | *A．capillaris* |
| 12 | 3 | 0.40 | Low | 0.0705 | 0.0708 | 1.0507 | *S． centauroides* |

**Table S2** Initial litter chemical characteristics of each species (Mean ± SE, the significant differences (*P* < 0.05) among different species are shown by different letters).

| Species | Carbon (C) content（%） | Nitrogen (N) content（%） | C /N ratio | Lignin content (g/g) | Cellulose content (g/g) |
| --- | --- | --- | --- | --- | --- |
| *Leymus chinensis* | 46.22±0.03^a^ | 1.91±0.02^a^ | 24.24±0.28^c^ | 0.16±0.00^b^ | 0.26±0.00^a^ |
| *Serratula centauroides* | 44.72±0.27^b^ | 1.47±0.07^b^ | 30.42±0.06^b^ | 0.31±0.01^a^ | 0.11±0.00^c^ |
| *Artemisia capillaris* | 46.76±0.30^a^ | 1.29±0.02^c^ | 36.39±0.40^a^ | 0.19±0.00^b^ | 0.20±0.00^b^ |

**Table S3** The z-scores of litter chemistry composition for each litter species.

| Species | Carbon (C) content | Nitrogen (N) content | C /N ratio | Lignin content | Cellulose content | Sum |
| --- | --- | --- | --- | --- | --- | --- |
| *Leymus chinensis* | 0.35 | -0.32 | 6.11 | 6.18 | -6.78 | 5.54 |
| *Serratula centauroides* | -0.08 | 1.18 | -0.07 | -8.85 | 8.09 | 0.25 |
| *Artemisia capillaris* | -0.27 | -0.86 | -6.04 | 2.68 | -1.3 | -5.80 |

**Table S4** The existing knowledge for the arrows in the priori model (Fig. 1 in the main text).

| Arrow | Theories used in the priori model |
| --- | --- |
| a | Litter evenness level can regulate the litter mass, C and N release of litter decomposition (Dickson & Wilsey, 2009; Swan et al., 2009; Zhang et al., 2020; Zhang et al., 2022). |
| b | Litter evenness level can affect the C/N of litter decomposition (Naeem et al., 2021). |
| c | Dominant species identity can regulate the mass loss, N release etc. on the processes of decomposition and affect C and nutrient cycle through litter decomposition (Dickson & Wilsey, 2009; Guo et al., 2022; Laird-Hopkins et al., 2017; Zhang et al., 2022). |
| d | There are significant differences in C/N between single and mixed decomposition of litter from different dominant specie (Asif et al., 2021). |
| e & f | The evenness of the species can quantify by community-weighted mean trait values (CWM; related to the mass ratio hypothesis) and different indices of functional diversity (FD; related to non-additive community effects) (Dias et al., 2013). |
| g & h | The dominant species can change the CWM and FDis of functional structure (Guo et al., 2022). |
| i & j | CWM can explain that litter decomposition resulted in differences in C concentration, N concentration, and C/N (Santonja et al., 2018) |
| k | FDis is one of the good predictors of litter N and C reserves and ecosystem total C reserves (Zhang et al., 2020). |
| l | FDis can explain some variations in litter decomposition due to significant differences in C/N (Santonja et al., 2018). |

**Table S5** Specific values of mass loss rate under different evenness level treatments on 69-day decomposition (Mean ± SE, the significant differences (*P* < 0.05) among different evenness levels are shown by different letters).

| Evenness level | Mass loss rate (%) |
| --- | --- |
| High | 59.26 ± 1.27^a^ |
| Medium | 58.08 ± 1.15^a^ |
| Low | 59.52 ± 1.62^a^ |
| Single species | 52.16 ± 2.15^b^ |

**Table S6** Specific values of mass loss rate, carbon (C) loss rate and nitrogen loss rate under different dominant species identity treatments on 30-, 69- and 127-day decomposition (Mean ± SE, the significant differences (*P* < 0.05) among different species are shown by different letters).

| Variable | Dominant species | *Leymus chinensis* | *Serratula centauroides* | *Artemisia capillaris* | *P-value* |
| --- | --- | --- | --- | --- | --- |
| **Mass loss rate** | 30-day decomposition | 41.424±1.659 | 38.427±2.679 | 40.687±2.450 | 0.636 |
|  | 69-day decomposition | 57.646±1.232 | 55.220±1.910 | 58.904±1.457 | 0.251 |
|  | 127-day decomposition | 57.270±1.644^b^ | 56.910±1.077^b^ | 61.056±1.535^a^ | **0.051** |
| **Carbon (C) loss rate** | 30-day decomposition | 48.033±1.325 | 44.212±2.732 | 47.533±2.345 | 0.425 |
|  | 69-day decomposition | 66.646±0.940 | 68.032±1.434 | 67.208±1.013 | 0.695 |
|  | 127-day decomposition | 70.920±0.854 | 72.778±1.474 | 72.193±1.250 | 0.551 |
| **Nitrogen (N) loss rate** | 30-day decomposition | 26.248±2.268 | 25.920±3.371 | 26.397±1.725 | 0.991 |
|  | 69-day decomposition | 38.474±1.984^b^ | 47.730±1.896^a^ | 40.553±1.906^b^ | **0.005** |
|  | 127-day decomposition | 38.630±2.221 | 44.678±3.278 | 42.062±2.560 | 0.302 |

**References list**

Asif T, Naeem I, Bu ZJ, Mallik A, Ma JZ, Rochefort L (2021) Litter mixing effects on decomposition in a peatland partially drained 30 years ago. Wetlands Ecology and Management 29: 883-895. <https://doi.org/10.1007/s11273-021-09818-4>

Biodiversity and tallgrass prairie decomposition: the relative importance of species identity, evenness, richness, and microtopography. Plant Ecology 201: 639-649. <https://doi.org/10.1007/s11258-008-9567-y>

Dias ATC, Berg MP, de Bello F, Van Oosten AR, Bílá K, Moretti M (2013) An experimental framework to identify community functional components driving ecosystem processes and services delivery. Journal of Ecology 101: 29-37. <https://doi.org/10.1111/1365-2745.12024>

Guo Q, Wen ZM, Ghanizadeh H, Zheng C, Fan YM, Yang X, et al. (2022) Shift in microbial communities mediated by vegetation-soil characteristics following subshrub encroachment in a semi-arid grassland. Ecology Indicators 137. <https://doi.org/10.1016/j.ecolind.2022.108768>

Laird-Hopkins BC, Brechet LM, Trujillo BC, Sayer EJ ( 2017) Tree functional diversity affects litter decomposition and arthropod community composition in a tropical forest. Biotropica 49: 903-911. <https://doi.org/10.1111/btp.12477>

Naeem I, Asif T, Wu XF, Hassan N, Liu YM, Wang HJ, et al. (2021) Species diversity induces idiosyncratic effects on litter decomposition in a Degraded Meadow Steppe. Frontiers in Environmental Science 9. <https://doi.org/10.3389/fenvs.2021.582409>

Santonja M, Foucault Q, Rancon A, Gauquelin T, Fernandez C, Baldy V, et al. (2018) Contrasting responses of bacterial and fungal communities to plant litter diversity in a Mediterranean oak forest. Soil Biology & Biochemistry 125: 27-36. <https://doi.org/10.1016/j.soilbio.2018.06.020>

Swan CM, Gluth MA, Horne CL (2009) Leaf litter species evenness influences nonadditive breakdown in a headwater stream. Ecology 90: 1650-1658. <https://doi.org/10.1890/08-0329.1>

Zhang XH, Wang L, Jiang W, Mao R (2020) Functional identity and functional diversity co-regulate litter mixture decomposition and nitrogen release in boreal riparian forest ponds. Biogeochemistry 151: 99-111. <https://doi.org/10.1007/s10533-020-00716-0>

Zhang XH, Zhang YH, Jiang SS, Song CC, Zhang JB, Mao R (2022) Dominant species and evenness level co-regulate litter mixture decomposition in a boreal peatland. Plant and Soil 474: 423-436. <https://doi.org/10.1007/s11104-022-05346-z>
